# Community support group intervention to promote self-management of non-communicable disease in Nepal: A pilot study

**DOI:** 10.1371/journal.pgph.0005941

**Published:** 2026-02-23

**Authors:** Rabina Dhakal, Barsha Thapa, Dibya Laxmi Manandhar, Buna Bhandari, Devika Rai, Supriya Shrestha, Sujata Sapkota

**Affiliations:** 1 Apara Innovations, Kathmandu, Nepal; 2 Manmohan Memorial Institute of Health Sciences, Kathmandu, Nepal; 3 Division of Health Sciences, Dwyer School of Health Sciences, Indiana University South Bend, Indiana, United States of America; 4 Central Department of Public health, Tribhuvan University Institute of Medicine, Kathmandu, Nepal; 5 Central Department of Population Studies, Tribhuvan University, Kathmandu, Nepal; Institute of Public Health Bengaluru, INDIA

## Abstract

Community support group interventions promote self-help, empower individuals to manage diseases and can be implemented in diverse formats. Their modality and effectiveness are influenced by broader local contexts. This pilot study aimed to explore whether conducting regular community support group meetings amongst people living with hypertension and/or diabetes, as a strategy to promote their self-management behaviours is feasible in Nepali setting. We conducted a mixed-method pilot study among adults aged ≥ 40 years living with hypertension and/or type 2 diabetes. Participants were enrolled into four groups–elderly-, marginalized-, mixed- and women’s-group—that met biweekly for three months to discuss self-management practices. We recorded all meeting discussions. Changes in weight, blood pressure, diet, and exercise were assessed using quantitative pre- and post-intervention surveys. Additionally, we conducted in-depth interviews with purposively selected participants and female community health volunteers who supported the intervention. Meeting discussions and interviews were analysed thematically. Integrated analysis of the quantitative and qualitative findings informed feasibility. Assessment of acceptability, demand, practicality and implementation was guided by Bowen’s framework. The study demonstrates community support group meetings as potentially feasible approach to promote self-management practices in people living with hypertension or diabetes. Over 80% of the total participants enrolled completed the study. Most expressed satisfaction with the meetings and reported that the meetings were beneficial and needed. Participation in these meetings also led to noticeable changes in self-management behaviours, particularly, dietary practices. Blood pressure and weight improved post-intervention. Members in homogeneous groups also initiated some collaborative self-management practices, such as going for walks together. To be sustainable, participants felt that such meetings should be led preferably by a resource person. Our findings suggest that regular, lifestyle-focused support group meetings can enhance NCD self-management. Homogenous group composition and expert facilitation may improve both engagement and long-term sustainability.

## 1. Introduction

Type 2 diabetes (T2D) and hypertension (HTN) are amongst the most prevalent non-communicable diseases (NCDs). Globally an estimated 33.0% of the adults aged 30–79 years are affected by HTN [1] and 10.5% of adults aged 20–79 years have T2D [2]. T2D and HTN pose significant risk to general health, increases the risks of other NCDs and adds to the socio-economic burden. The burden imposed on Low- and Middle-Income Countries (LMICs), including Nepal, is staggering [3]. T2D and HTN prevalence in Nepal is reported at 8.5% and 24.5% respectively [4,5].

NCDs, including T2D and HTN, can be prevented and effectively managed using lifestyle modifications to address common risk factors, such as unhealthy dietary practices, physical inactivity, and consumption of tobacco and alcohol [6–8]. However, making sustained modifications to the lifestyle is not easy. Strategies to promote lifestyle modification and NCD self-management continue to evolve and vary between contexts and settings [9–14].

Community-based interventions are increasingly being used and tested for NCD management and prevention [9–21]. These interventions have employed different approaches including interventions that are led by professionals, such as healthcare workers, trained community health volunteers [13,14,21], or allied health professionals [15], or interventions that are led by non-professionals, for example, those engaging family members, friends, peers [22] and the people affected by the disease themselves [17]. Although inconsistent, existing evidence suggest that both professional-led and non-professional-led interventions can have positive impacts on health and behavioural outcomes [18–21,23].

Interventions that provide social support—through non-professionals like family members, friends, peers as well as through professionals—have shown to improve clinical outcomes, promote healthy lifestyles, and reduce psychosocial symptoms among adults with diabetes [18–21,23]. Similarly, self-help–based interventions that empower individuals with diabetes or hypertension through structured training and mobilization into peer groups have demonstrated benefits for health and well-being [24,25].

Such community-based support strategies delivered through peer leaders, family members, affected individuals, community health workers, or healthcare professionals have proven feasible in several settings including diabetes support group in the USA [19], Accredited Social Health Activists (ASHA) led community support groups in India [13], self-help groups in Indonesia [10] and trained lay peer leaders in Kerala, India [22]. This indicates that community-based, support-oriented interventions for NCD management are heterogeneous in structure, leadership, and mode of delivery, and can be tailored to different health system capacities and contextual settings.

In Nepal, community-based self-help group intervention in leprosy programs have reduced stigma and improve overall well-being [20]. Similarly, mobilization of female community health volunteers (FCHVs) has contributed to improved blood pressure control and stroke care [21,23]. However, concerns remain regarding sustainability, as FCHVs are already overburdened and Nepal faces a critical shortage of health workers [24,25]. These constraints highlight the need for alternative, community-empowering approaches to expand NCD support.

Based on this premises, we conducted this pilot study to explore whether regular community support group (CSG) meetings conducted amongst patients with HTN and/or T2D as a strategy to promote their self-management behaviours is feasible in Nepali setting. Specifically, the study aimed to explore participants’ interest and acceptability of such meetings, their preferences regarding the modalities of support group delivery, and the potential of these meetings to support self-management behaviours.

## 2. Materials and methods

We conducted a pilot intervention study guided by Bowen’s framework [26] to explore the feasibility of community support group meetings to promote NCD self-management. The study employed a mixed-method research design with a qualitative priority approach [27,28].

### 2.1. Setting

The study was conducted in Kageshwori Manohara Municipality (ward-6) in Kathmandu district in Nepal. Intervention and data collection was conducted between March – August 2023. The baseline data collection was started on May 3, 2023 and the endline data collection completed on 19 July 2023.

### 2.2. Participants and recruitment

We recruited adults aged ≥40 years with a confirmed diagnosis of HTN and/or T2D residing in Kageshwori Manohara Municipality-6 who were able to care for themselves. Individuals with complications, requiring frequent or urgent medical attention or enrolled in other interventional studies were excluded. As a pilot study focused on understanding intervention feasibility acceptability, as well as viable modality, participant numbers (sample size) were based on practical considerations of group sizes for effective group meetings. We did not perform a formal sample size calculation.

Participants were recruited with the support from four Female Community Health Volunteers (FCHVs) providing services in the ward. Each FCHV was requested to identify 8–12 individuals living in close proximity who could be organized into specific support groups:

- Elderly group (criteria: men and women aged ≥ 60 years),
- Marginalized group (criteria: men and women identifying themselves as *Dalit*- an ethnic category classified under marginalised group by Nepal government [29])
- Women groups (criteria: women participants) and,
- Mixed groups (criteria: both men and women participants)

The FCHVs collaborated to identify and engage potential participants from their catchment areas, categorizing them into the specified groups. They approached potential participants, explained the study purpose and procedures, and invited them to an orientation session.

### 2.3. Intervention

Broadly, the intervention involved forming four community support groups of individuals sharing similar needs and socio-demographic characteristics and conducting support group meetings on a fortnightly basis to discuss experiences and learnings about the disease and (self-)management. The meetings served as a platform for participants to share and learn from each other’s experiences regarding lifestyle-related healthy practices for the self-management of HTN and T2D. The meetings were facilitated by a research team member.

### 2.4. Process

#### Orientation sessions.

We invited participants identified by the FCHVs to an orientation session. Orientation session was conducted separately for two groups and in three sessions for: 1) women’s and mixed groups, 2) elderly group, and 3) marginalised group. Orientation for the elderly was held closer to their homes due to mobility challenges. We arranged separate sessions for the marginalised group in response to an observed reluctance among some participants to integrate across social groups.

Research team members—all trained and working in the NCD management sector—facilitated the orientation sessions. They were supported by a research assistant. Participants received detailed information on HTN and T2D, covering various approaches to prevention and management, with an emphasis on lifestyle practices such as diet, physical activity, alcohol and tobacco use, medication adherence, monitoring, and foot care. The facilitators also detailed the objective of the research, proposed research process, concept behind support groups, how the support groups were expected to function, what was expected of the group members and ethical aspects, including their right to leave the study at any time without adverse consequences. Discussions were held and those who consented to participate were formally enrolled into the study. Baseline data was collected following the orientation session after taking informed written consent.

#### Formation of community support groups.

The participants identified by FCHVs for inclusion in each category, who attended orientation sessions and consented to the study, as described above, were formally assigned to their designated groups.

#### Community support group meetings.

Two weeks after orientation sessions, we commenced community support group (CSG) meetings. The meetings were held on a fortnightly basis, separately for each group. FCHVs coordinated with group members to schedule meeting times and venues, with support from the research assistant as needed. Each group held five meetings during the study period. The first four meetings focused on specific topics: 1) dietary practices, 2) physical activity, 3) medicines use and adherence and 4) monitoring and foot-care; and, in the fifth meeting a collective discussion was held. The fifth meeting facilitated collective reflection and gathered participant feedback. Research team members facilitated all sessions, guiding discussions, addressing misconceptions, and encouraging peer support. Each meeting began with a recap of the previous session and a discussion of any changes participants had made. All meetings were audio-recorded.

### 2.5. Outcomes

Feasibility assessment was guided by Bowen’s feasibility framework [26]. Of the eight areas of focus proposed by the framework, we assessed feasibility based on four parameters: a) acceptability, b) demand, c) practicality and d) implementation [26]. The indicators used for each were adapted for the study (Table 1).

**Table 1:** Feasibility assessment indicators.

	Feasibility Indicators	Indicators assessment standards	Assessment method
A)	Acceptability To what extent is the intervention judged satisfying, or attractive to participants?	• Meeting participation and intent to continue • Satisfaction	Quantitative: Participants retention in the study and meeting attendance Qualitative: Assessment of satisfaction and perceived appropriateness
B)	Demand To what extent is the intervention needed and actually used to modify self-management behaviours?	• Programs need and, perceived demand • Estimated or actual use of the program (including initiation of self-management practices)	Qualitative: Assessment of reported gaps in self-management behaviours and practices; participants view on the need of the meetings, and assessment of changes or attempts to modify self-management behaviours during implementation period
C)	Practicality How is the practical aspect of the intervention (resources, and circumstances, meeting modalities) perceived?	• Views on intervention modalities (meeting moderation, meeting time, group composition, content)	Qualitative: Group members and FCHVs views on meeting modalities (Meeting Discussion/ IDI); and researchers’ impressions of the modalities
D)	Implementation To what extent is the intervention? implemented/effective and what impacted implementation?	• Preliminary impact of the intervention (effectiveness evaluation) • Factors affecting implementation ease or difficulty	Quantitative: Preliminary impact assessment Qualitative: Assessment of Meeting discussions

### 2.6. Data collection

#### Qualitative data.

The qualitative component was the key method for assessing feasibility parameters and involved:

Group Meetings: We audio-recorded all CSG meeting discussions with the permission of participants. Additionally, at each meeting, the research assistant took notes to capture participants’ engagement.

In-depth interviews: Post-intervention–a month after the fifth meeting—we conducted in-depth interviews (IDIs) with purposively selected participants (n = 16; 4 from each group), and with the FCHVs (n = 4) to explore their perceptions. The interviews focused on understanding participants’ and FCHVs’ views about CSG meetings, their experiences and perceived feasibility (focusing on feasibility indicators) and usefulness of meetings in NCD self-management, barriers and facilitators of conducting the meetings and preferred modality. In-depth interview protocol guided these interviews. All interviews were audio recorded.

#### Quantitative data.

Baseline and end-line information were collected using a pre-designed structured questionnaire, developed specifically for the study. In addition to participants’ socio-demographic characteristics, the questionnaire included information on weight, blood pressure and their self-management (diet, physical activity, medications, alcohol and smoking) practices. The research assistant collected data on participants’ demography and behavioural practices and measured weight, while a trained health worker from the Municipal Hospital measured blood pressure. Participants meeting attendance data was also collected. Quantitative data were collected to complement the qualitative findings and to assess potential impact of the intervention on clinical outcomes and self-management behaviour.

### Ethical consideration

The study received ethical approval from the Nepal Health Research Council (Reg number: 146/2023). All ethical requirements were fulfilled, and participants at each step were fully informed about the study objectives and their right to participate or withdraw without any repercussions. Both verbal and written informed consent were obtained. All study activities were conducted in close collaboration with local stakeholders.

### 2.7. Data analysis

#### Qualitative data.

The meeting discussions and interview recordings were transcribed verbatim. The transcripts and the meeting notes were analysed using a thematic analysis approach. Two researchers coded each transcript. Coding employed an inductive approach as a first step. The initial list of codes was then discussed between the researchers. Once the consensus was reached on the emerging themes, they were categorized into broader categories, deductively to fit into the framework indicators.

#### Quantitative data.

Data collected at baseline and endline were analysed using SPSS 23. Descriptive statistics were calculated to assess the change in dietary practices and physical activity. T-test was performed to measure the statistical significance observed in the continuous variables such as blood pressure and weight.

Data integration occurred primarily at the analysis stage. Feasibility parameters were assessed through the combined analysis of qualitative and quantitative data, as outlined in Table 1.

## 3. Results

Of the 44 individuals who attended the orientation, 41 were enrolled in the study. Eight were enrolled in the women’s group, 12 in elderly group, 11 in marginalised and 10 in the mixed group. Elderly group members were all female.

### 3.1. Participants’ demographics and self-management behaviours

Eighty percent of the enrolled participants were female. The mean age of participants was 61 years (range 40–84 years). Almost three-fourths of the participants had never attended formal school; and only 9.8% had completed secondary education. Majority identified themselves as Brahmin/Chettri (68.3%), followed by Dalit (26.8%) and Janajati (4.9%). (Table 2).

**Table 2:** Participants socio-demographic characteristics.

Characteristics	Number (Percentage)	Mean (SD)
**Age group (age in years) (N = 41)**		
40- 50	7 (17.1)	61 (11.67)
51-60	14 (34.1)	
61-70	10 (24.4)	
> 70	10 (24.4)	
**Gender (N = 41)**		
Female	33 (80.5)	
Male	8 (19.5)	
**Ethnicity (N = 41)**		
Brahmin/ Chettri	28 (68.3)	
Dalit	11 (26.8)	
Janjati	2 (4.9)	
**Education Status (N = 41)**		
Illiterate	26 (63.4)	
Non-formal education	5 (12.2)	
Primary (1–5 class)	4 (9.8)	
Lower secondary (67–8)	1 (2.4)	
Secondary	4 (9.8)	
Bachelor and above	1 (2.4)	
**Family Type (N = 41)**		
Nuclear	17 (41.5)	
Joint	24 (58.5)	

The majority (>80%) reported they neither smoked nor consumed alcohol. Approximately 85.3% of the participants were taking medications for HTN; and 80.0% for diabetes. Sixty-five percent reported they exercised regularly (Table 3).

**Table 3:** Self-management behaviours at baseline.

Characteristics	Number (Percentage)
**Smoking (N = 41)**	
Yes	7 (17.1)
No	34 (82.9)
**Alcohol consumption (N = 41)**	
Yes	5 (12.2)
No	36 (87.8)
**Regular exercise (N = 41)**	
Yes	27 (65.9)
No	14 (34.1)
**On medication for diabetes (N = 20)**	
Yes	16 (80.0)
No	4 (20.0)
**On medication for hypertension (N = 34)**	
Yes	29 (85.3)
No	5 (14.7)

### 3.2. Intervention feasibility

Our study demonstrates that conducting CSG meetings is generally feasible as an intervention to promote NCD self-management in a semi-urban Nepali setting. Over 80% of participants were retained throughout the pilot study period, and most expressed satisfaction with the meetings, valuing the discussions and advice on diet, medication adherence, and lifestyle modifications. Participation in these meetings also resulted in noticeable changes in self-management behaviours, particularly in dietary practices. Additionally, the quantitative data at the endline showed improvement in blood pressure. While both participants and FCHVs found meeting modalities mostly appropriate, marginalised group participants reported difficulty in managing time for the meetings. Participants felt that to be effective and sustainable, such meetings should be led by an expert- someone who could guide the group. Groups by themselves, participants felt, would not be effective and sustainable.

#### A) Acceptability.

**Meeting participation and intent to continue group meetings**

Participant retention and meeting attendance indicated satisfactory outcomes, suggesting participants’ intention and willingness to regularly participate in the group meetings.

Of the total enrolled, 82.9% completed the study. Furthermore, most participants in all groups attended all the meetings during the study period. The overall meeting attendance rate was 82.3%. The lowest attendance (70%) was recorded for mixed group participants. Women’s group had the highest attendance rate (90%). All enrolled participants attended the first CSG meeting in their respective groups. (Table 4)

**Table 4:** Meeting attendance by groups (N = 41).

Group	Orientation Meeting	Community Support Group Meetings				
		1^st^	2^nd^	3^rd^	4^th^	5^th^
Women	8 (19.5%)	7 (87.5%)	7 (87.5%)	6 (75.0%)	7 (87.5%)	7 (87.5%)
Elderly	12 (29.2%)	10 (83.3%)	11 (91.6%)	12 (100%)	9 (75.0%)	11(91.6%)
Marginalised	11 (26.8%)	10 (90.9%)	10 (90.9%)	11 (100%)	9 (81.8%)	7 (63.6%)
Mixed	10 (24.4%)	5 (50.0%)	9 (90.0%)	7 (70.0%)	4 (40.0%)	9 (90.0%)
Total:	41 (100%)	32(78.0%)	37(90.2%)	37(90.2%)	29(70.7%)	34(82.9%)

**Satisfaction**

Participants expressed satisfaction with the CSG meetings, citing benefits such as improved knowledge, increased confidence, strengthened group bonds, and motivation to modify self-management behaviours.

*“I liked the meetings, and I am happy because the meetings reminded me to take a balanced diet and medicines regularly. I have been suffering a lot since I used to miss my medicines frequently” (IDI, Elderly group participant).*

*“Whenever I cook food in my home, I remember the discussions of these meetings about limiting the use of salt and oil. I am so happy that these meetings alerted me to the required lifestyle behaviours” (Meeting Discussion, Mixed group participant).*

*“After attending the meeting, I am very much confident that I can motivate other people with similar diseases of my community during causal meetings” (Meeting discussion, Marginalised group participant)*

Meetings also became a platform to meet and mingle with friends and was perceived as a good usage of time.

*“When I come to the meeting, I can meet my friends, talk and have fun (in addition to) discussing our problems, individual practices and preferences for promoting healthy habits. I get to spend my leisure time in a fruitful way.” (Meeting discussions, Elderly group participant)*

The FCHVs who were closely involved in the meetings also expressed their satisfaction and usefulness of such meetings in the community- both to the participants and to themselves.

*“(because of these meetings), I could meet them (people) more frequently, share and discuss health-related information including diabetes and hypertension, and I am very satisfied that my bond with people has grown better” (IDI, FCHV).*

#### B) Demand.

**Program need and perceived demand**

General difficulties or participants’ neglect regarding self-management practices were evident in the meeting discussions, underscoring the need for platforms that can provide support for their disease management efforts.

*“I know that walking everyday early in the morning will benefit my health, but in the absence of a person who could accompany me, I have not been able to practice walking regularly. Someone should be there to encourage and insist us to do so. Now some of us can go (for walks) together.” (Meeting discussion, Women’s group participant)*

*“Once I was told in a health camp that my blood glucose level had gone up and was asked to go for a confirmatory test, but I never went” (Meeting discussion, Marginalised group participant)*

Both participants and FCHVs reported the need for such program, not only for patients but also for the broader community and emphasized that it should be continued for a longer duration.

*“For me, it would be good if you could continue the meetings at a frequent interval of time and include more people from our community. I hope this group meeting will continue for a longer period.” (IDI, Mixed group participant)*

*“Most participants (I’ve interacted with) have said that the meetings have been useful. They’ve asked me to continue such meetings once every 1 to 2 months. These meetings have prompted self-management practices such as diet control and physical activity” (IDI, FCHV)*

Participants found that the meetings provided them with more time to discuss dietary and physical activity than they would get during medical consultations.

*“Last month, I went for my check up at the hospital. The doctor was very busy, and I couldn’t ask about my health properly during that meeting. Here, you discuss diet, physical activity and others and it has been very useful for me.” (Meeting discussion, Mixed group participant)*

**Estimated or actual use of the program**

In the meetings, participants openly discussed their self-management behaviour, shared concerns and looked for insights and experience from fellow- group members. The meetings raised participants’ interest in collaborative self-management activities. Some initiated changes in their existing self-management practices. Participants felt that the meetings were a good reminder call for healthy behaviours and raised interest in collaborative activities.

Following the CSG meetings, participants began to implement several changes in their NCD self-management behaviours, particularly in dietary practices. While dietary modifications were primarily reported by participants in the mixed and women’s groups, some members of the marginalized group also indicated that they had started reducing their carbohydrate intake. Participants from the three groups, excluding the marginalized group, reported an increase in their consumption of fruits and vegetables.

*“I used to eat fried bitter gourds and lady’s fingers, but after coming to the meeting, I learned from the group that it can be boiled and prepared as a salad. I started consuming it as a salad, and it tastes well, too.” (Meeting Discussion, Marginalised group participant)*

Participants from all, except the marginalised group, reported increased consumption of fruits and vegetables and consistent efforts to reduce salt and oil intake.

Furthermore, participants also reported initiating yoga and “brief walk” with increased consistency in physical activities for those already engaged. A few members of the mixed and women’s group formed a group and went for morning and evening walks together. A participant from a marginalised group even shared that she started walking after the first group meeting and had lost approximately three kilograms in three weeks. A few reported increased consistencies in physical activities.

*“(Before) I thought daily household tasks were enough to manage my blood sugar. However, I’ve learned that incorporating yoga and other exercises is essential. I now walk at least an hour a day, and yesterday, my blood sugar level was in control.” (Meeting discussion, Women group participant)*

Group members motivated each other for regular check-ups and monitoring of blood glucose and HTN. Even those reluctant to go to health facilities went for check-up. In some cases, a new diagnosis was confirmed.

*“After attending the meetings, I went for a check-up, got tested and was diagnosed with diabetes. I have started taking medicine” (Meeting discussion, Mixed group participant)*

Participants also made efforts to take their medications regularly.

*“……..It’s the little things we learned here, you told us that you keep medicine under your pillow so that you don’t forget………ask your grandchild to schedule alarm in his mobile, he will then remind you to take the medicine”. (Meeting Discussion, Elderly group participant)*

#### C) Practicality.

**Meeting modality**

**a. Need for an expert to lead the meetings**

Participants strongly voiced that for support group meetings to be effective and sustainable, the meetings should be led by an expert – a resource person. They suggested that expert-led meetings would be more fruitful, expressing their concerns about sustaining regular meetings without an expert. Participants were reluctant to the idea of one of them or a community member leading and facilitating such meetings. They also highlighted the possibility of misinformation without expert guidance. Each meeting was led by a member of the research team; and participants felt that this modality worked. They stated that without someone from “outside” to “lead” the sessions, people “may not take the meetings seriously”. While a few members, mostly from women and marginalised group expressed interest in leading/initiating activities such as physical training exercises to engage the community, they felt that only their efforts and initiation would be insufficient to sustain these activities.

*“I have been asking the members of this group multiple times to start doing physical exercise daily in our community. But no one follows me since I do not have the required skills to keep them engaged.” (Meeting Discussion, Marginalised group participant)*

Furthermore, participants felt that occasional visits by a doctor or allied health professional and a provision of blood glucose and HTN monitoring would add value to these meetings.

*“These meetings are very nice, but if we had visits from nurses or doctors, having our sugar and pressure tested, along with getting the right information, would be an added value.” (Meeting discussion, Elderly group participant)*

**b. Group composition**

Conducting meetings in homogeneous groups consisting of members from within their own community and sharing common demographic characteristics, such as: gender, age, ethnicity and place of residence interests helped participants better relate to the discussions.

*“I feel comfortable in the meeting because we are similar people from our own community, and we relate ourselves with our experiences. You can form this kind of group in other places too” (Meeting discussion, Marginalised group participant)*

Nonetheless, one FCHV contemplated the possible advantages of including ‘younger’ people in the group for varied perspectives, diverse knowledge and experience sharing.

*“I think when there were people sharing similar backgrounds in the group, they would have the same thoughts, experiences and problems. If we had mixed the younger people with the elderly group, there would be more discussions. The elderly would have learned from younger people’s knowledge, and younger ones would have learned from their experiences.” (IDI, FCHV)*

**c. Meeting times**

The meetings were scheduled in consultation with the participants. Therefore, most participants were able to participate in the meetings. However, a few participants from marginalised groups, especially wage labourers, had difficulty managing time, even when the meetings were scheduled on Saturdays.

*“I tried to convince people not to miss any meetings, but they have their priorities. So, they were reluctant to come to the meetings.” (Meeting discussion, Marginalised group participant)*

**d. Meeting contents**

The research team conceived the meeting modalities and the content to be covered in each meeting. Participants were generally happy with the content. More interest and enthusiasm were seen around diet and physical activity management – making these the topics most (enthusiastically) discussed. Nevertheless, discussion on foot care drew attention, especially among those who had T2D, given that they had the least knowledge in the area of foot care. Foot care, we identified as a topic that was least discussed in general and during care consultations.

#### D) Implementation.

**Preliminary impact of intervention:**

The baseline and endline assessment indicated positive changes in blood pressure, weight and diet practices. While the small sample size precludes firm conclusions, the results indicate the potential for behaviour change among patients following participation in the meetings.

**a. Effect on blood pressure and weight**

Significant reductions were noted in the systolic and diastolic pressure of the participants after the intervention (Table 5). There was a decrease in systolic blood pressure by 16.7 points (t = 3.8, CI = 7.76, 25.80, p=<0.01) and in diastolic blood by 5.7 points (t = 2.3, CI = 0.71, 10.71, p = 0.02) at the endline compared to those at the baseline assessment. Significant changes were also noted in the weight of participants after the intervention. (Table 5)

**Table 5:** Changes in blood pressure and weight.

Parameters	Mean	Mean Diff.	CI	T	p-value
Systolic Pressure Baseline (n = 28)	142.1	16.7	7.76, 25.80	3.8	<0.001
Systolic Pressure Endline (n = 28)	125.3				
Diastolic Pressure Baseline (n = 28)	86.7	5.7	0.71, 10.71	2.3	0.027
Diastolic Pressure Endline (n = 28)	81.1				
Weight Baseline (n = 30)	59.7	0.6	0.04, 1.09	2.2	0.035
Weight Endline (n = 30)	59.1				

**b. Effect on dietary practices and physical activity**

Although statistically non-significant, improvements in dietary habits as well as physical activity were observed after the intervention. (Table 6) The percentage of participants who reported taking three or more small meals increased from 31.7% to 38.2%. Similarly, daily consumption of fruits increased remarkably from 24.4% to 47.1%; and those who reported consuming salt four times daily decreased from 4.9% to 0%. Participants who reported performing some form of exercise increased by almost 13%. (Table 6)

**Table 6:** Changes in dietary patterns of the participants.

Parameter	Baseline (N = 41) Number (Percentage)	Endline (N = 34) Number (Percentage)
**Dietary behaviour**		
Meals per day		
One meal	6 (14.6)	2 (5.9)
Two meals	22 (53.7)	19 (55.9)
Three meals	10 (24.4)	10 (29.4)
More than three meals	3 (7.3)	3 (8.8)
**Salt per day**		
Two times	29 (70.7)	25 (73.5)
Three times	10 (24.4)	9 (26.5)
Four times	2 (4.9)	0.0
**Ghee**		
Daily	6 (14.6)	4 (11.8)
2–3 times a week	1 (2.4)	
Sometimes	10 (24.4)	10 (29.4)
Do not consume	24 (58.5)	20 (58.8)
**Daily consumption of fruits**		
Yes	10 (24.4)	16 (47.1)
No	31 (75.6)	18 (52.9)
**Physical Activity**		
Yes	27 (65.9)	27 (79.4)
No	14 (34.1)	7 (20.6)

Data disaggregated for each group is presented in S1 File.

**Factors affecting implementation**

A range of factors impacted intervention implementation. Involvement of the FCHVs from the outset of the intervention allowed effective engagement, participant identification and helped to successful implementation. Similarly, engaging a health worker from the municipal hospital (to measure blood pressure) and support from local level stakeholders (municipality hospital, local stakeholders, including mayor, ward-chairs and members) helped researchers gain community’s trust and facilitated the study process. Availability of the community hall and other public places like the temple’s premises, with space for meetings allowed the meetings to be conducted in proximity that were closer to the participants. On the other hand, despite interest, wage labour and household activities challenged timely scheduling of the meetings. Due to the health emergency/COVID 19 resurgence in the country during the study period, few meetings of elderly groups had to be halted. The barriers and facilitators to effective implementation are listed in Table 7.

**Table 7:** Facilitators and barriers to implementation.

Facilitators (What made implementation easy?)	Barriers (What made implementation difficult?)
• Accessible meeting venues and appropriate timing • Support from Municipality office, Municipal Hospital, FCHVs and health workers • Self-awareness of the disease/condition on the need of dietary modifications and physical activity • Self determination to make lifestyle changes • Family support has been crucial for all participants, especially for elderly group • Perceived threat of the consequences of disease outcome • Consequences of not adhering to the treatment procedure, regular follow-up visits leading to the medical emergency requiring surgery among relatives and few participants	• Occupied with work and household chores, especially for the marginalized group, and the women participants from mixed group and women’s group • Health emergencies like COVID 19 hamper the regular attendance of the meetings • Social events -difficulty in managing time for the meeting as well as maintaining consistent lifestyle modifications (the women participants from mixed group and women’s group • Perception that T2D/HTN does not improve despite self-management efforts. • Felt lack of decision-making power in the family (especially in women participants) • Compromised self-care ability (people with disabilities and elderly)

## 4. Discussion

Our study demonstrates that regular community support group meetings is an acceptable and a potentially feasible and effective approach to manage NCD self-management behaviours among people living with T2D and/or HTN in Nepali setting. The study provides valuable insights on the modality and potential mechanisms of community support group intervention for promoting HTN and T2D self-management behaviours. In designing community support group meetings to promote NCD management, our study highlights that a) group composition, b) meeting contents and c) facilitation are the components that need utmost consideration.

Support groups are fundamentally designed to include individuals facing similar issues, such as, diabetes, hypertension and cancer [9,10,13,19,30,31]. While the formation of support groups based on disease condition and/or on a specific issue/problem is essential, our study points to the need of considering group homogeneity based on added characteristics, such as age, gender and day-to-day activities to promote and encourage the discussion around NCD self-management behaviours. For example, in our study, women’s group and elderly group (all female) participants were more consistently present in the meetings compared to participants of the marginalised and mixed group. Although other factors could certainly have played a role in their ability to join the meetings - the discussions showed that participants from these groups could relate better in their everyday experience, were more comfortable and willing to share experiences with each other. As they knew each other from before and belonged to the same community they were able to offer support beyond the meetings. In the case of marginalised groups and mixed group participants, which were more diverse in terms of demography, such as age and gender - cohesion and practical support beyond the meetings appeared more difficult.

The content covered in the meetings significantly influenced participants’ enthusiasm and engagement. Each meeting session was structured to focus on specific self-management practice, with diet management receiving the most attention. Participants showed a strong interest in dietary management behaviours, frequently asking questions about healthy dietary practices and the challenges of adopting healthier eating habits. Notably, they were actively implementing changes to their dietary practices more than other self-management behaviours. This indicates not only an interest in diet management, but also diet/ food as practice that people have awareness of the need for change but find making consistent change challenging. An important point to consider here is also that our majority participants were female- a group that is a ‘socio-cultural in-charge’ of preparing food for the household [32]. People also encounter practical challenges to effective diet management [33]. Our study shows that participants are keen on finding ways to navigate these challenges to maintain health. Discussions that guide the ways will attract people’s attention and interest.

Regarding content, our study also highlights foot-care as a neglected aspect and the need to pay attention to this aspect in everyday diabetes management. Lack of adequate focus on foot-care aspect of diabetes self-management and the need for foot-care education and timely referral and management has been previously reported [34,35]. A recent study also highlights the need of determining the most effective health promotion model and implementation strategy to enhance foot care among diabetic patients [36].

For acceptability and effectiveness of the meetings, our study shows expert-led sessions are preferred. Participants expected someone with expertise to guide the sessions. While peer support—defined as the provision of emotional, informational, and appraisal support by individuals with lived experience—has demonstrated value in chronic disease management [37], our findings suggest that, in this context, peer-led activities alone may be insufficient to ensure sustainability and optimal impact. Participants highlighted the importance of involving an expert or resource person to support and guide these activities over the long term. Concerns were also raised about the potential risk of misinformation in the absence of expert oversight, particularly if meeting content is not carefully structured and monitored. The concern stems from a range of myths surrounding diabetes and HTN management in Nepal [33,38] and participants desire to learn the ‘right’ information. From alternative therapy to fear of harm from ‘English medicines’, a range of misinformation circulates in the Nepali community about NCDs [33,38].

Furthermore, participants in our study said that provision of regular monitoring of blood pressure and blood glucose at these meetings would create added value. While this comes with cost implications, incorporating such facilities would certainly enhance participation. This is supported by an open cluster randomised trial conducted in China where village doctor-led interventions resulted in the positive improvements in the blood pressure control [39].

This intervention is innovative and, to our knowledge, the first to evaluate the use of group meetings as a strategy for promoting NCD self-management in the Nepali context. It establishes a strong case for implementing a community-based intervention for self-management of hypertension and diabetes. The feasibility, including positive preliminary impact of lowering blood pressure, weight and improving lifestyle practices, although encouraging – the essential next step is to explore this further to establish the evidence on effectiveness and sustainability. Furthermore, cost analysis despite being an important component of any intervention, we did not undertake a formal cost evaluation in this study. Nonetheless, our experience suggests that such meetings can be implemented at a reasonable cost. The primary cost components for an expert-led model, as preferred by participants, would include the expert’s time, the involvement of FCHVs and health staff as appropriate (costed according to local rates), and the provision of basic refreshments during meetings. In low-resource settings such as Nepal, where health system budgets are constrained, understanding these cost implications is critical for assessing scalability and sustainability. Future studies should therefore incorporate formal cost and cost-effectiveness analyses to inform policy and programme scale-up. This study was designed as a pilot feasibility investigation, conducted over a short period with a small number of participants. Its primary aim was to generate proof of concept regarding the intervention’s feasibility, delivery modality, acceptability, and potential impact. Given its exploratory nature, no formal sample size calculation was performed. While the findings provide initial insights, they should be interpreted with caution, and larger, adequately powered studies are needed to confirm these results. Larger trials conducted over longer duration and diverse settings are further needed, particularly to establish effectiveness, scalability and sustainability.

## 5. Conclusion

Community support group meetings, held on regular basis, to discuss lifestyle practices are promising community-based interventions in Nepal for NCD self-management. Meetings conducted with the homogenous group members and guided by an expert are likely to be more effective. Larger trials are needed to establish scalability and long-term effectiveness.

## Supporting information

S1 File Supporting information. Disaggregated results.
(DOCX)

S2 File Data File. Supplementary qualitative quotes.
(DOCX)

S3 File Data File. Individual level quantitative raw data.
(XLSX)

## References

[1] WHO. Global report on hypertension: the race against a silent killer. 2023. https://www.who.int/publications/i/item/9789240081062

[2] International Diabetes Federation. IDF Diabetes Atlas. Brussels: International Diabetes Federation. 2021. https://diabetesatlas.org/atlas/tenth-edition/

[3] IHME. Global Burden of Disease 2021: Findings from the GBD 2021 Study. 2021.https://www.healthdata.org/research-analysis/library/global-burden-disease-2021-findings-gbd-2021-study

[4] Dhimal M, Bista B, Bhattarai S, Dixit Prasai L, Hyder MKA, Agrawal N. Noncommunicable Disease Risk Factors: STEPS Survey Nepal 2019. 2019.

[5] Shrestha N, Karki K, Poudyal A, Aryal KK, Mahato NK, Gautam N, et al. Prevalence of diabetes mellitus and associated risk factors in Nepal: findings from a nationwide population-based survey. BMJ Open. 2022;12(2):e060750. doi: 10.1136/bmjopen-2022-060750 35193925 PMC8867329

[6] Appel LJ. Lifestyle modification as a means to prevent and treat high blood pressure. J Am Soc Nephrol. 2003;14(7 Suppl 2):S99–102. doi: 10.1097/01.asn.0000070141.69483.5a 12819311

[7] Uusitupa M, Khan TA, Viguiliouk E, Kahleova H, Rivellese AA, Hermansen K, et al. Prevention of Type 2 Diabetes by Lifestyle Changes: A Systematic Review and Meta-Analysis. Nutrients. 2019;11(11):2611. doi: 10.3390/nu11112611 31683759 PMC6893436

[8] WHO. Hypertension. https://www.who.int/news-room/fact-sheets/detail/hypertension

[9] Isiguzo GC, Santo K, Panda R, Mbau L, Mishra SR, Ugwu CN, et al. Adherence Clubs to Improve Hypertension Management in Nigeria: Clubmeds, a Feasibility Study. Glob Heart. 2022;17(1):21. doi: 10.5334/gh.1109 35342700 PMC8932363

[10] Maryam R, Sahar J, Pudjiati A, Prayetni A, Ekasari M, Rosidawati A. Self Help Group Activity in Improving Knowledge On Elderly with Hypertension in Jakarta, Indonesia. Indian Journal of Public Health Research & Development. 2019;10:1935.

[11] McPake B. The need for cost-effective and affordable responses for the global epidemic of non-communicable diseases. The Lancet Global Health. 2019;7(10):e1293–4. 10.1016/S2214-109X(19)30375-4 31537346

[12] Ni Z, Atluri N, Shaw RJ, Tan J, Khan K, Merk H, et al. Evaluating the Feasibility and Acceptability of a Mobile Health-Based Female Community Health Volunteer Program for Hypertension Control in Rural Nepal: Cross-Sectional Study. JMIR Mhealth Uhealth. 2020;8(3):e15419. doi: 10.2196/15419 32149712 PMC7091025

[13] Riddell MA, Mini GK, Joshi R, Thrift AG, Guggilla RK, Evans RG, et al. ASHA-Led Community-Based Groups to Support Control of Hypertension in Rural India Are Feasible and Potentially Scalable. Front Med (Lausanne). 2021;8:771822. doi: 10.3389/fmed.2021.771822 34881267 PMC8645590

[14] Tan J, Xu H, Fan Q, Neely O, Doma R, Gundi R, et al. Hypertension Care Coordination and Feasibility of Involving Female Community Health Volunteers in Hypertension Management in Kavre District, Nepal: A Qualitative Study. Glob Heart. 2020;15(1):73. doi: 10.5334/gh.872 33150138 PMC7583706

[15] Krishnan A, Ekowati R, Baridalyne N, Kusumawardani N, Suhardi, Kapoor SK, et al. Evaluation of community-based interventions for non-communicable diseases: experiences from India and Indonesia. Health Promot Int. 2011;26(3):276–89. doi: 10.1093/heapro/daq067 21071458

[16] Xiao N, Long Q, Tang X, Tang S. A community-based approach to non-communicable chronic disease management within a context of advancing universal health coverage in China: progress and challenges. BMC Public Health. 2014;14 Suppl 2(Suppl 2):S2. doi: 10.1186/1471-2458-14-S2-S2 25082410 PMC4120154

[17] Parasuraman G, Jeemon P, Thankappan KR, Ali MK, Mahal A, McPake B. Community control of hypertension and diabetes (CoCo-HD) program in the Indian states of Kerala and Tamil Nadu: a study protocol for a type 3 hybrid trial. BMC Public Health. 2024;24:2275. 39169312 10.1186/s12889-024-19746-6 PMC11340170

[18] WHO. Trinidad and Tobago: empowering communities to prevent and self-manage noncommunicable diseases - PAHO/WHO | Pan American Health Organization. 2021. https://www.paho.org/en/stories/trinidad-and-tobago-empowering-communities-prevent-and-self-manage-noncommunicable-diseases

[19] Gilden JL, Hendryx MS, Clar S, Casia C, Singh SP. Diabetes support groups improve health care of older diabetic patients. J Am Geriatr Soc. 1992;40(2):147–50. doi: 10.1111/j.1532-5415.1992.tb01935.x 1740599

[20] Jay S, Winterburn M, Choudhary R, Jha K, Sah AK, O’Connell BH, et al. From social curse to social cure: A self‐help group community intervention for people affected by leprosy in Nepal. Community & Applied Soc Psy. 2021;31(3):276–87. doi: 10.1002/casp.2510

[21] Basnet B, Yadav JK, Gajurel BP, Shing YK, Kandel B, Nepal G. Role of female community health volunteers in ischemic stroke prevention, identification, referral and rehabilitation in Nepal. Ann Med Surg (Lond). 2021;72:102893. doi: 10.1016/j.amsu.2021.102893 34992775 PMC8712991

[22] Thankappan KR, Sathish T, Tapp RJ, Shaw JE, Lotfaliany M, Wolfe R, et al. A peer-support lifestyle intervention for preventing type 2 diabetes in India: A cluster-randomized controlled trial of the Kerala Diabetes Prevention Program. PLoS Med. 2018;15(6):e1002575. doi: 10.1371/journal.pmed.1002575 29874236 PMC5991386

[23] Neupane D, McLachlan CS, Mishra SR, Olsen MH, Perry HB, Karki A, et al. Effectiveness of a lifestyle intervention led by female community health volunteers versus usual care in blood pressure reduction (COBIN): an open-label, cluster-randomised trial. Lancet Glob Health. 2018;6(1):e66–73. doi: 10.1016/S2214-109X(17)30411-4 29241617

[24] Wasti SP, van Teijlingen E, Rushton S, Subedi M, Simkhada P, Balen J, et al. Overcoming the challenges facing Nepal’s health system during federalisation: an analysis of health system building blocks. Health Res Policy Syst. 2023;21(1):117. doi: 10.1186/s12961-023-01033-2 37919769 PMC10621174

[25] Khatri RB, Mishra SR, Khanal V. Female Community Health Volunteers in Community-Based Health Programs of Nepal: Future Perspective. Front Public Health. 2017;5:181. doi: 10.3389/fpubh.2017.00181 28785555 PMC5519587

[26] Bowen DJ, Kreuter M, Spring B, Cofta-Woerpel L, Linnan L, Weiner D, et al. How we design feasibility studies. Am J Prev Med. 2009;36(5):452–7. doi: 10.1016/j.amepre.2009.02.002 19362699 PMC2859314

[27] Wisdom JP, Cavaleri MA, Onwuegbuzie AJ, Green CA. Methodological reporting in qualitative, quantitative, and mixed methods health services research articles. Health Serv Res. 2012;47(2):721–45. doi: 10.1111/j.1475-6773.2011.01344.x 22092040 PMC3419885

[28] Designing and Conducting Mixed Methods Research. https://collegepublishing.sagepub.com/products/designing-and-conducting-mixed-methods-research-3-241842

[29] NSO. National Population and Housing Census 2021 National Report on Caste/Ethnicity, Language & Religion. GoN. 2021. https://censusnepal.cbs.gov.np/results/files/result-folder/Caste%20Ethnicity_report_NPHC_2021.pdf

[30] Fisher EB, Boothroyd RI, Elstad EA, Hays L, Henes A, Maslow GR, et al. Peer support of complex health behaviors in prevention and disease management with special reference to diabetes: systematic reviews. Clin Diabetes Endocrinol. 2017;3:4. doi: 10.1186/s40842-017-0042-3 28702258 PMC5471959

[31] Tehrani AM, Farajzadegan Z, Rajabi FM, Zamani AR. Belonging to a peer support group enhance the quality of life and adherence rate in patients affected by breast cancer: A non-randomized controlled clinical trial. J Res Med Sci. 2011;16(5):658–65. 22091289 PMC3214378

[32] Komatsu H, Malapit HJL, Theis S. Does women’s time in domestic work and agriculture affect women’s and children’s dietary diversity?. Food Policy. 2018;79:256–70.

[33] Sapkota S, Brien J-AE, Gwynn J, Flood V, Aslani P. Perceived impact of Nepalese food and food culture in diabetes. Appetite. 2017;113:376–86. doi: 10.1016/j.appet.2017.03.005 28288801

[34] Matricciani L, Jones S. Who cares about foot care? Barriers and enablers of foot self-care practices among non-institutionalized older adults diagnosed with diabetes: an integrative review. Diabetes Educ. 2015;41(1):106–17. doi: 10.1177/0145721714560441 25480398

[35] Xu X, Zheng S, Cao Z, Jiang H, Shi L, Wang Z, et al. Evaluation of diabetic foot care knowledge, determinants of self‐care practices and the efficacy of health education. International Wound Journal. 2024;21(2). doi: 10.1111/iwj.14704 PMC10850605 42052900

[36] Wong Y-T, Morrison SC. Health Promotion Models for Improving Footcare in Older Adults: A Scoping Review. Gerontology. 2024;70(8):785–800. doi: 10.1159/000538868 38636462 PMC11309045

[37] Thompson DM, Booth L, Moore D, Mathers J. Peer support for people with chronic conditions: a systematic review of reviews. BMC Health Serv Res. 2022;22(1):427. doi: 10.1186/s12913-022-07816-7 35361215 PMC8973527

[38] Sapkota S, Brien JE, Aslani P. Nepalese patients’ perceptions of treatment modalities for type 2 diabetes. Patient Prefer Adherence. 2016;10:1777–86. 27695296 10.2147/PPA.S113467 PMC5028164

[39] Sun Y, Mu J, Wang DW, Ouyang N, Xing L, Guo X. A village doctor-led multifaceted intervention for blood pressure control in rural China: an open, cluster randomised trial. The Lancet. 2022;399(10339):1964–75. 10.1016/S0140-6736(22)00325-7 35500594

