# Supplementary material for: Community support group intervention to promote self-management of non-communicable disease in Nepal: A pilot study
Source: PLOS Glob Public Health. 2026 Feb 23;6(2):e0005941. doi: 10.1371/journal.pgph.0005941 (PMC12928488; doi:10.1371/journal.pgph.0005941)
Supplement: S1 File — Disaggregated results. (DOCX) [file pgph.0005941.s001.docx]

**Table A: Demographic distribution of the CSG members based on type of group**

| **Parameters** | **Elderly Group (N=11)** | **Women's Group (N=9)** | **Mixed Group (N=10)** | **Marginalized Group (N=11)** |
| --- | --- | --- | --- | --- |
| **Caste** |  |  |  |  |
| Brahmin/Chhetri | 11 (100%) | 9 (100%) | 8 (80%) |  |
| Dalit |  |  |  | 11 (100%) |
| Janajati |  |  | 2 (20%) |  |
| **Gender** |  |  |  |  |
| Female | 11 (100%) | 9 (100%) | 8 (80%) | 5 (45.5%) |
| Male |  |  | 2 (20%) | 6 (54.5%) |
| **Age Group** |  |  |  |  |
| 40-50 |  | 2 (22.2%) | 3 (30%) | 2 (18.2%) |
| 51-60 |  | 4 (44.4%) | 3 (30%) | 6 (54.5%) |
| 61-70 | 5 (45.5%) | 2 (22.2%) | 3 (30%) | 1 (9.1%) |
| above 70 | 6 (54.5%) | 1 (11.1%) | 1 (10%) | 2 (18.2%) |
| **Education Status** |  |  |  |  |
| Illiterate | 8 (72.7%) | 5 (55.6%) | 6 (60%) | 7 (63.6%) |
| Non-formal education | 2 (18.2%) |  | 1 (10%) | 2 (18.2%) |
| Primary (1-5 class) | 1 (9.1%) | 1 (11.1%) | 1 (10%) | 1 (9.1%) |
| Lower secondary (6-8) |  |  |  | 1 (9.1%) |
| Secondary |  | 3 (33.3%) | 1 (10%) |  |
| Bachelors and above |  |  | 1 (10%) |  |

**Table B: Changes in dietary patterns of the participants according to the type of group**

| **Group** |  | **Elderly** | | **Women** | | **Mixed** | | **Marginalized** | |
| --- | --- | --- | --- | --- | --- | --- | --- | --- | --- |
| **Parameters** |  | **Baseline (N=11)** | **Endline (N=9)** | **Baseline (N=9)** | **Endline (N=7)** | **Baseline (N=10)** | **Endline (N=8)** | **Baseline (N=11)** | **Endline (10)** |
| Dietary Behaviour | |  |  |  |  |  |  |  |  |
| Meals per day | One meal | 3(27.3%) | 2(22.2%) | 1(11.1%) |  |  |  | 2(18.2%) |  |
|  | Two meals | 5(45.4%) | 1(11.1%) | 3(33.3%) | 7(100%) | 7(70%) | 3(37.5%) | 7(63.6%) | 8(80%) |
|  | Three meals | 3(27.3%) | 5(55.6%) | 2(22.2%) |  | 3(30%) | 3(37.5%) | 2(18.2%) | 2(20%) |
|  | More than three meals |  | 1(11.1%1) | 3(33.3%) |  |  | 2(25%) |  |  |
| Salt per day | Two times | 10(90.9%) | 8(88.9%) | 5(55.6%) | 6(85.7%) | 8(80%) | 7(87.5%) | 6(54.5%) | 4(40%) |
|  | Three times | 1(9.1%) | 1(11.1%) | 3(33.3%) | 1(14.3%) | 2(20%) | 1(12.5%) | 4(36.4%) | 6(60%) |
|  | Four times |  |  | 1(11.1%) |  |  |  | 1(9.1%) |  |
| Ghee | Daily | 3(27.3%) | 2(22.2%) | 1(11.1%) |  | 2(20%) | 1(12.5%) |  | 1(10%) |
|  | 2-3 times a week |  |  |  |  |  |  | 1(9.1%) |  |
|  | Sometimes | 2(18.2%) | 6(66.7%) | 5(55.6%) | 3(42.9%) | 1(10%) | 1(12.5%) | 2(18.2%) |  |
|  | Do not consume | 6(54.5%) | 1(11.1%) | 3(33.3%) | 4(57.1%) | 7(70%) | 6(75%) | 8(72.7%) | 9(90%) |
| Daily consumption of fruits | Yes | 3(27.3%) | 6(66.7%) | 5(55.6%) | 7(100) | 2(20%) | 3(37.5%) |  |  |
|  | No | 8(72.7%) | 3(33.3%) | 4(44.4%) |  | 8(80%) | 5(62.5%) | 11(100%) | 10(100%) |
| Physical Activity | Yes | 5(45.45) | 7(77.8%) | 6(66.7%) | 6(85.7%) | 7(70%) | 6(75%) | 9(81.8%) | 8(80%) |
|  | No | 6(54.5%) | 2(22.2%) | 3(33.3%) | 1(14.3%) | 3(30%) | 2(25%) | 2(18.2%) | 2(20%) |
