# Supplementary material for: Community support group intervention to promote self-management of non-communicable disease in Nepal: A pilot study
Source: PLOS Glob Public Health. 2026 Feb 23;6(2):e0005941. doi: 10.1371/journal.pgph.0005941 (PMC12928488; doi:10.1371/journal.pgph.0005941)
Supplement: S2 File — Supplementary qualitative quotes. (DOCX) [file pgph.0005941.s002.docx]

S2 File

Different themes/subthemes on the feasibility assessment

| Themes | Sub-theme | Supporting Verbatim |
| --- | --- | --- |
| Acceptability | Satisfaction | I liked the meetings, and I am happy because the meetings reminded me to take a balanced diet and medicines regularly. I have been suffering a lot since I used to miss my medicines frequently (IDI - Elderly group participant).  Whenever I cook food in my home, I remember the discussions of these meetings about limiting the use of salt and oil. I am so happy that these meetings alerted me to the required lifestyle behaviours (Meeting Discussion, Mixed group participant).  After attending the meeting, I am very much confident that I can motivate other people with similar diseases of my community during causal meetings (Meeting Discussion, Marginalised group participant)  When I come to the meeting, I can meet my friends, talk and have fun (in addition to) discussing our problems, individual practices and preferences for promoting healthy habits. I get to spend my leisure time in a fruitful way. (Meeting Discussion, Elderly group participant)  (because of these meetings), I could meet them (people) more frequently, share and discuss health-related information including diabetes and hypertension, and I am very satisfied that my bond with people has grown better (IDI, FCHV). |
| Demand | Program need and perceived demand | I know that walking everyday early in the morning will benefit my health, but in the absence of a person who could accompany me, I have not been able to practice walking regularly. Someone should be there to encourage and insist n us to do so. Now some of us can go (for walks) together. (Meeting Discussion, Women’s group participant)  Once I was told in a health camp that my blood glucose level had gone up and was asked to go for a confirmatory test, but I never went (Meeting Discussion, Marginalised group participant)  For me, it would be good if you could continue the meetings at a frequent interval of time and include more people from our community. I hope this group meeting will continue for a longer period. (IDI, Mixed group participant)  *Most participants (I’ve interacted with) have said that the meetings have been useful. They’ve asked me to continue such meetings once every 1 to 2 months. These meetings have prompted self-management practices such as diet control and physical activity (IDI, FCHV)*  Last month, I went for my check up at the hospital. The doctor was very busy, and I couldn’t ask about my health properly during that meeting. Here, you discuss diet, physical activity and others and it has been very useful for me. (Meeting Discussion, Mixed group participant) |
|  | Estimated or actual use of the program | I used to eat fried bitter gourds and lady's fingers, but after coming to the meeting, I learned from the group that it can be boiled and prepared as a salad. I started consuming it as a salad, and it tastes well, too. (Meeting Discussion, Marginalised group participant)  (Before) I thought daily household tasks were enough to manage my blood sugar. However, I've learned that incorporating yoga and other exercises is essential. I now walk at least an hour a day, and yesterday, my blood sugar level was in control. (Meeting Discussion, Women group participant)  After attending the meetings, I went for a check-up, got tested and was diagnosed with diabetes. I have started taking medicine (Meeting Discussion, Mixed group participant)  ……..It’s the little things we learned here, you told us that you keep medicine in your pillow so that you don’t forget………ask your grandchild to schedule alarm in his mobile, he will then remind you to take the medicine. (Meeting Discussion, Elderly group participant) |
| Practicality | Need for an expert led meeting | I have been asking the members of this group multiple times to start doing physical exercise daily in our community. But no one follows me since I do not have the required skills to keep them engaged. (Meeting Discussion, Marginalised group participant)  These meetings are very nice, but if we had visits from nurses or doctors, having our sugar and pressure tested, along with getting the right information, would be an added value. (Meeting Discussion, Elderly group participant) |
|  | Group Composition | I feel comfortable in the meeting because we are similar people from our own community, and we relate ourselves with our experiences. You can form this kind of group in other places too (Meeting Discussion, Marginalised group participant)  I think when there were people sharing similar backgrounds in the group, they would have the same thoughts, experiences and problems. If we had mixed the younger people with the elderly group, there would be more discussions. The elderly would have learned from younger people's knowledge, and younger ones would have learned from their experiences. (IDI- FCHV) |
|  | Meeting Times | I tried to convince people not to miss any meetings, but they have their priorities. So, they were reluctant to come to the meetings. (Meeting Discussion, Marginalised group participant) |
| Implementation | Factors affecting implementation  (Barriers) | *In the morning my daughter-in-law goes to work. So, I have to give them (grandchildren) breakfast, make her hair, then make food, make them eat and send them to school. Only after that I will have time to breathe. So, I don’t have time to walk in the morning. (Meeting Discussion, Marginalized group participant)*  *Cooking is a family affair – and family’s desire becomes important to own sometimes (Meeting Discussion, Women and Elderly group participant)*  *if you are taking 3-4 medicines the medicines will control – there is lesser need to focus on non-pharmacological measures (if you are on medicines – self-management measures are secondary) (Meeting Discussion, Elderly group)*  *If we start taking medicines, we have to take it for lifelong. It will cause many side effects such as kidney problems, gastritis. (Meeting Discussion, Mixed group participant)*  *There is no tendency to give much details about the medicine to the patients. (Meeting Discussion, Mixed group participant)*  *I am not comfortable asking a doctor about the clear details of medication. I fear he would judge me negatively (Meeting Discussion, Women’s group participant)* |
|  | Facilitators | *He (referring to someone the participant knew) was a patient of sugar. They had to cut his leg because he had a small wound and because of that small wound it went deeper and deeper. (Meeting Discussion, Marginalized group participant)*  *No, I haven't forgotten taking medication. If I tried to sleep without medication, my grandson reminds me; he says: “Even if you forget other things, don’t forget to take this medicine. (Meeting Discussion, Elderly Group participant)* |
